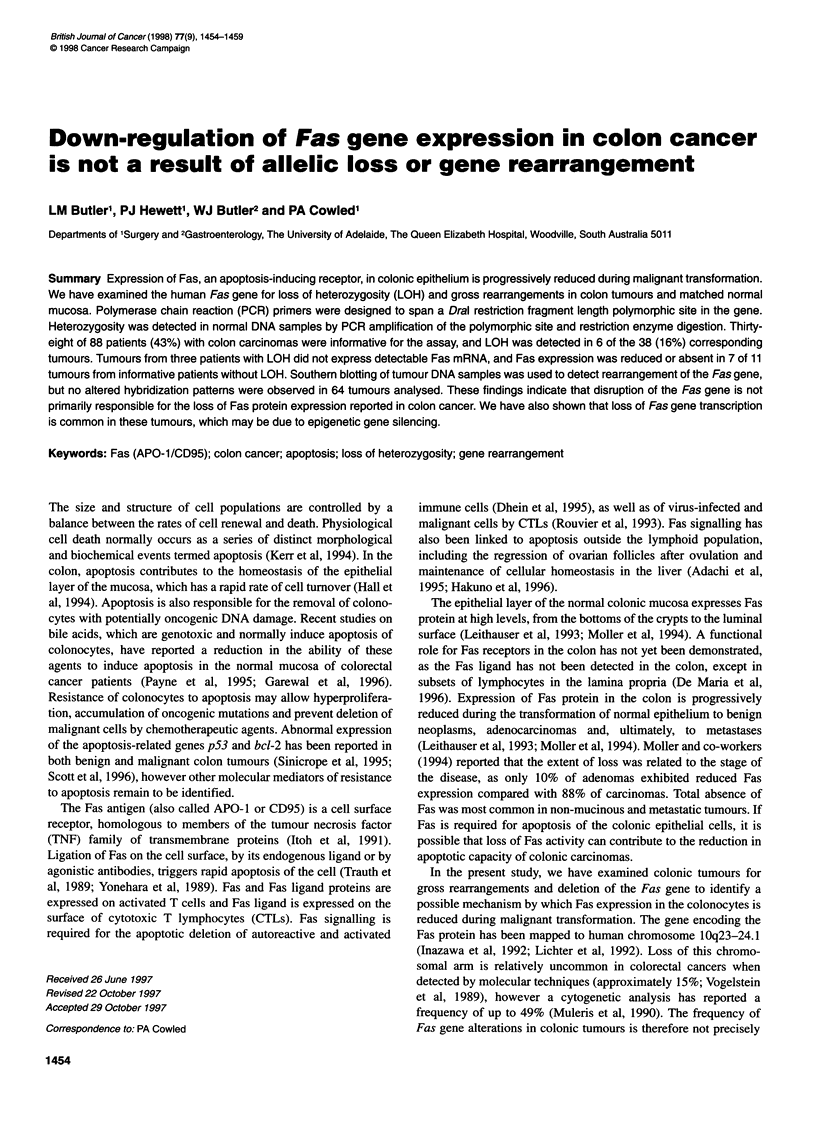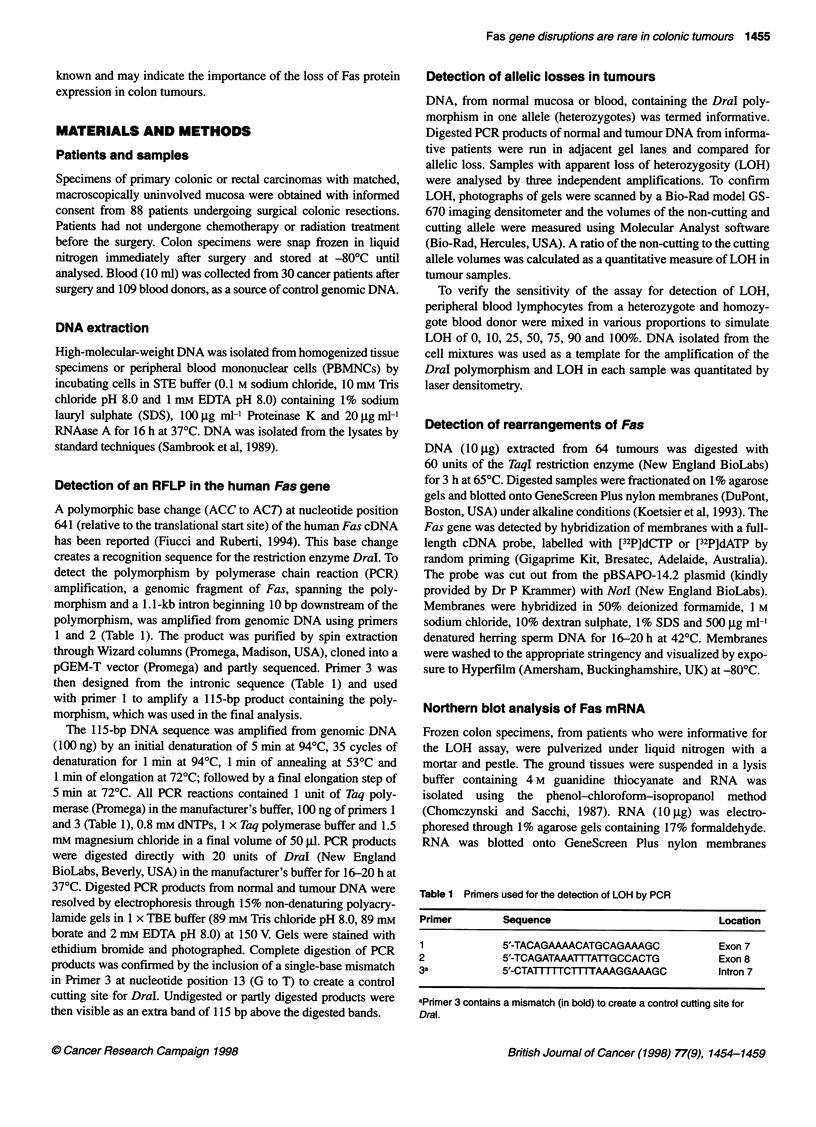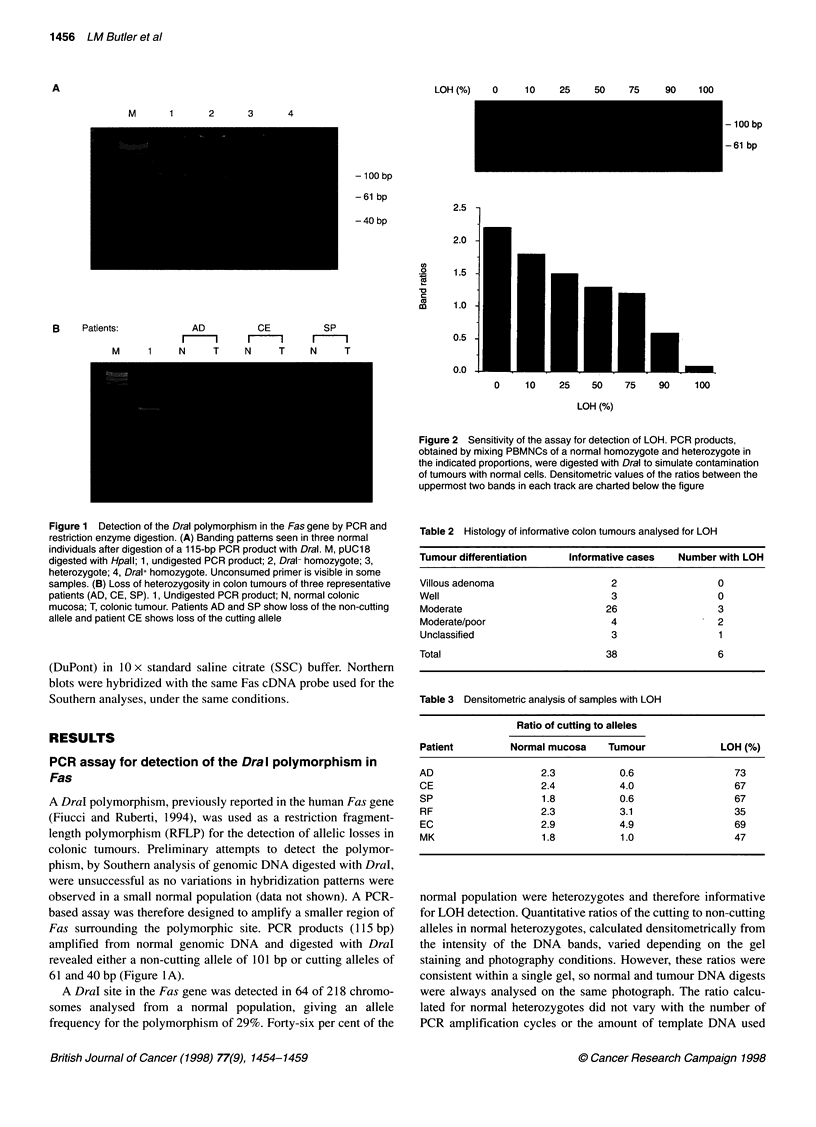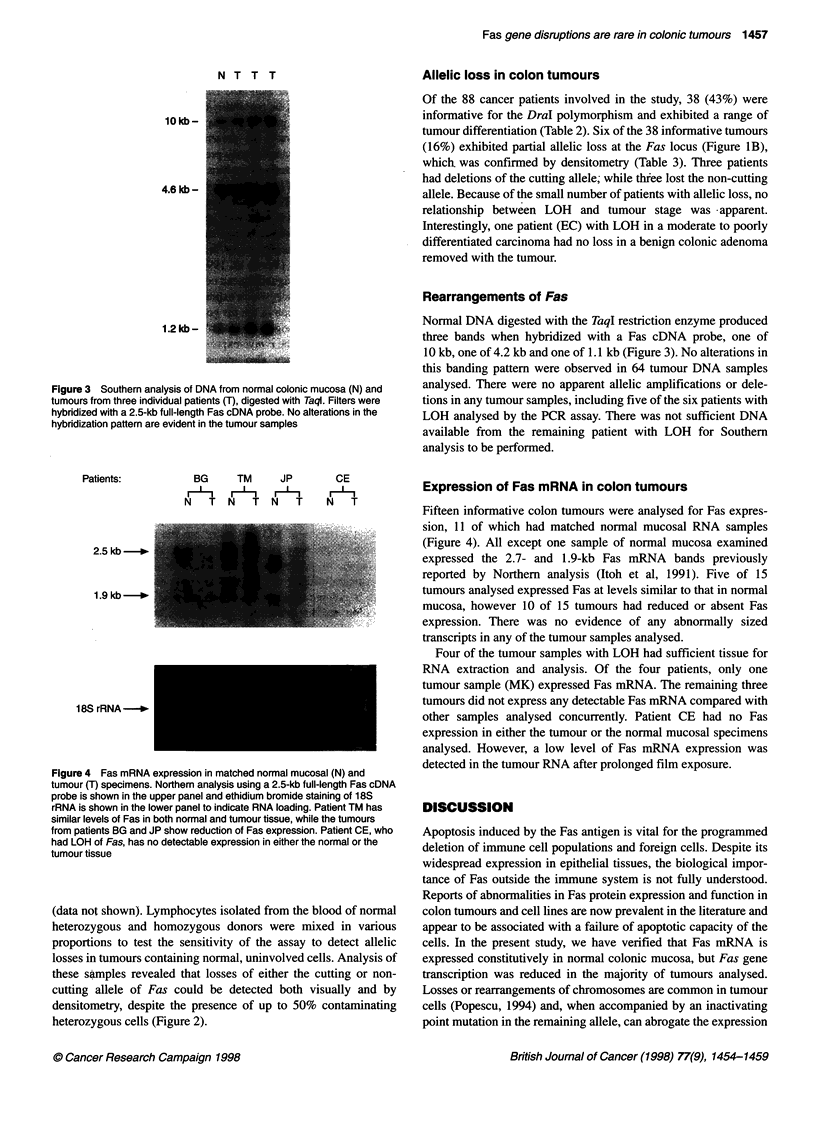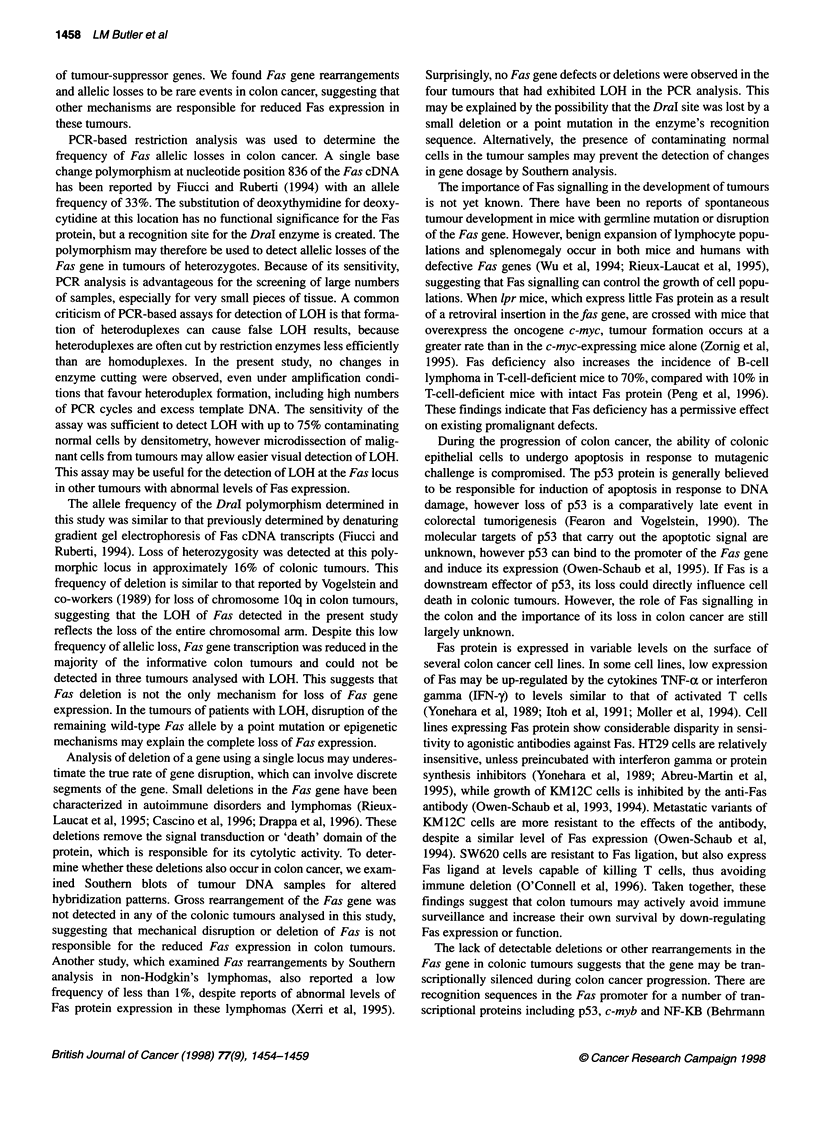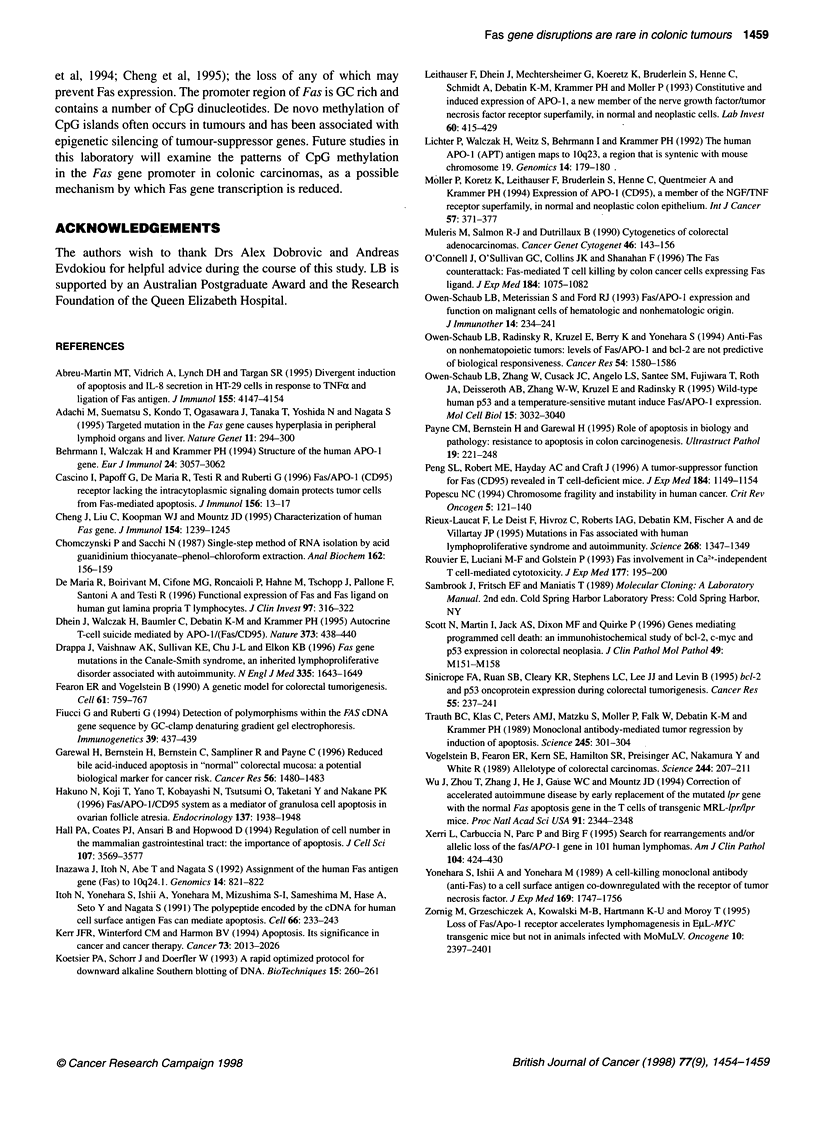# Down-regulation of Fas gene expression in colon cancer is not a result of allelic loss or gene rearrangement.

**DOI:** 10.1038/bjc.1998.239

**Published:** 1998-05

**Authors:** L. M. Butler, P. J. Hewett, W. J. Butler, P. A. Cowled

**Affiliations:** Department of Surgery, The University of Adelaide, The Queen Elizabeth Hospital, Woodville, South Australia.

## Abstract

**Images:**


British Journal of Cancer (1998) 77(9), 1454–1459
© 1998 Cancer Research Campaign

### Down-regulation of *Fas* gene expression in colon cancer is not a result of allelic loss or gene rearrangement

**LM Butler[1], PJ Hewett[1], WJ Butler[2] and PA Cowled[1]**

Departments of [1]Surgery and [2]Gastroenterology, The University of Adelaide, The Queen Elizabeth Hospital, Woodville, South Australia 5011

**Summary** Expression of Fas, an apoptosis-inducing receptor, in colonic epithelium is progressively reduced during malignant transformation. We have examined the human *Fas* gene for loss of heterozygosity (LOH) and gross rearrangements in colon tumours and matched normal mucosa. Polymerase chain reaction (PCR) primers were designed to span a *Dra*I restriction fragment length polymorphic site in the gene. Heterozygosity was detected in normal DNA samples by PCR amplification of the polymorphic site and restriction enzyme digestion. Thirty-eight of 88 patients (43%) with colon carcinomas were informative for the assay, and LOH was detected in 6 of the 38 (16%) corresponding tumours. Tumours from three patients with LOH did not express detectable Fas mRNA, and Fas expression was reduced or absent in 7 of 11 tumours from informative patients without LOH. Southern blotting of tumour DNA samples was used to detect rearrangement of the *Fas* gene, but no altered hybridization patterns were observed in 64 tumours analysed. These findings indicate that disruption of the *Fas* gene is not primarily responsible for the loss of Fas protein expression reported in colon cancer. We have also shown that loss of *Fas* gene transcription is common in these tumours, which may be due to epigenetic gene silencing.

**Keywords:** Fas (APO-1/CD95); colon cancer; apoptosis; loss of heterozygosity; gene rearrangement

The size and structure of cell populations are controlled by a balance between the rates of cell renewal and death. Physiological cell death normally occurs as a series of distinct morphological and biochemical events termed apoptosis (Kerr et al, 1994). In the colon, apoptosis contributes to the homeostasis of the epithelial layer of the mucosa, which has a rapid rate of cell turnover (Hall et al, 1994). Apoptosis is also responsible for the removal of colonocytes with potentially oncogenic DNA damage. Recent studies on bile acids, which are genotoxic and normally induce apoptosis of colonocytes, have reported a reduction in the ability of these agents to induce apoptosis in the normal mucosa of colorectal cancer patients (Payne et al, 1995; Garewal et al, 1996). Resistance of colonocytes to apoptosis may allow hyperproliferation, accumulation of oncogenic mutations and prevent deletion of malignant cells by chemotherapeutic agents. Abnormal expression of the apoptosis-related genes *p53* and *bcl-2* has been reported in both benign and malignant colon tumours (Sinicrope et al, 1995; Scott et al, 1996), however other molecular mediators of resistance to apoptosis remain to be identified.

The Fas antigen (also called APO-1 or CD95) is a cell surface receptor, homologous to members of the tumour necrosis factor (TNF) family of transmembrane proteins (Itoh et al, 1991). Ligation of Fas on the cell surface, by its endogenous ligand or by agonistic antibodies, triggers rapid apoptosis of the cell (Trauth et al, 1989; Yonehara et al, 1989). Fas and Fas ligand proteins are expressed on activated T cells and Fas ligand is expressed on the surface of cytotoxic T lymphocytes (CTLs). Fas signalling is required for the apoptotic deletion of autoreactive and activated immune cells (Dhein et al, 1995), as well as of virus-infected and malignant cells by CTLs (Rouvier et al, 1993). Fas signalling has also been linked to apoptosis outside the lymphoid population, including the regression of ovarian follicles after ovulation and maintenance of cellular homeostasis in the liver (Adachi et al, 1995; Hakuno et al, 1996).

The epithelial layer of the normal colonic mucosa expresses Fas protein at high levels, from the bottoms of the crypts to the luminal surface (Leithauser et al, 1993; Moller et al, 1994). A functional role for Fas receptors in the colon has not yet been demonstrated, as the Fas ligand has not been detected in the colon, except in subsets of lymphocytes in the lamina propria (De Maria et al, 1996). Expression of Fas protein in the colon is progressively reduced during the transformation of normal epithelium to benign neoplasms, adenocarcinomas and, ultimately, to metastases (Leithauser et al, 1993; Moller et al, 1994). Moller and co-workers (1994) reported that the extent of loss was related to the stage of the disease, as only 10% of adenomas exhibited reduced Fas expression compared with 88% of carcinomas. Total absence of Fas was most common in non-mucinous and metastatic tumours. If Fas is required for apoptosis of the colonic epithelial cells, it is possible that loss of Fas activity can contribute to the reduction in apoptotic capacity of colonic carcinomas.

In the present study, we have examined colonic tumours for gross rearrangements and deletion of the *Fas* gene to identify a possible mechanism by which Fas expression in the colonocytes is reduced during malignant transformation. The gene encoding the Fas protein has been mapped to human chromosome 10q23–24.1 (Inazawa et al, 1992; Lichter et al, 1992). Loss of this chromosomal arm is relatively uncommon in colorectal cancers when detected by molecular techniques (approximately 15%; Vogelstein et al, 1989), however a cytogenetic analysis has reported a frequency of up to 49% (Muleris et al, 1990). The frequency of *Fas* gene alterations in colonic tumours is therefore not precisely

*Received 26 June 1997*
*Revised 22 October 1997*
*Accepted 29 October 1997*

*Correspondence to:* PA Cowled known and may indicate the importance of the loss of Fas protein expression in colon tumours.

### MATERIALS AND METHODS

### Patients and samples

Specimens of primary colonic or rectal carcinomas with matched, macroscopically uninvolved mucosa were obtained with informed consent from 88 patients undergoing surgical colonic resections. Patients had not undergone chemotherapy or radiation treatment before the surgery. Colon specimens were snap frozen in liquid nitrogen immediately after surgery and stored at –80°C until analysed. Blood (10 ml) was collected from 30 cancer patients after surgery and 109 blood donors, as a source of control genomic DNA.

### DNA extraction

High-molecular-weight DNA was isolated from homogenized tissue specimens or peripheral blood mononuclear cells (PBMNCs) by incubating cells in STE buffer (0.1 M sodium chloride, 10 mM Tris chloride pH 8.0 and 1 mM EDTA pH 8.0) containing 1% sodium lauryl sulphate (SDS), 100 μg $ml^{-1}$ Proteinase K and 20 μg $ml^{-1}$ RNAase A for 16 h at 37°C. DNA was isolated from the lysates by standard techniques (Sambrook et al, 1989).

### Detection of an RFLP in the human *Fas* gene

A polymorphic base change (AC*C* to AC*T*) at nucleotide position 641 (relative to the translational start site) of the human *Fas* cDNA has been reported (Fiucci and Ruberti, 1994). This base change creates a recognition sequence for the restriction enzyme *Dra*I. To detect the polymorphism by polymerase chain reaction (PCR) amplification, a genomic fragment of *Fas*, spanning the polymorphism and a 1.1-kb intron beginning 10 bp downstream of the polymorphism, was amplified from genomic DNA using primers 1 and 2 (Table 1). The product was purified by spin extraction through Wizard columns (Promega, Madison, USA), cloned into a pGEM-T vector (Promega) and partly sequenced. Primer 3 was then designed from the intronic sequence (Table 1) and used with primer 1 to amplify a 115-bp product containing the polymorphism, which was used in the final analysis.

The 115-bp DNA sequence was amplified from genomic DNA (100 ng) by an initial denaturation of 5 min at 94°C, 35 cycles of denaturation for 1 min at 94°C, 1 min of annealing at 53°C and 1 min of elongation at 72°C; followed by a final elongation step of 5 min at 72°C. All PCR reactions contained 1 unit of *Taq* polymerase (Promega) in the manufacturer's buffer, 100 ng of primers 1 and 3 (Table 1), 0.8 mM dNTPs, 1 × *Taq* polymerase buffer and 1.5 mM magnesium chloride in a final volume of 50 μl. PCR products were digested directly with 20 units of *Dra*I (New England BioLabs, Beverly, USA) in the manufacturer's buffer for 16–20 h at 37°C. Digested PCR products from normal and tumour DNA were resolved by electrophoresis through 15% non-denaturing polyacrylamide gels in 1 × TBE buffer (89 mM Tris chloride pH 8.0, 89 mM borate and 2 mM EDTA pH 8.0) at 150 V. Gels were stained with ethidium bromide and photographed. Complete digestion of PCR products was confirmed by the inclusion of a single-base mismatch in Primer 3 at nucleotide position 13 (G to T) to create a control cutting site for *Dra*I. Undigested or partly digested products were then visible as an extra band of 115 bp above the digested bands.

### Detection of allelic losses in tumours

DNA, from normal mucosa or blood, containing the *Dra*I polymorphism in one allele (heterozygotes) was termed informative. Digested PCR products of normal and tumour DNA from informative patients were run in adjacent gel lanes and compared for allelic loss. Samples with apparent loss of heterozygosity (LOH) were analysed by three independent amplifications. To confirm LOH, photographs of gels were scanned by a Bio-Rad model GS-670 imaging densitometer and the volumes of the non-cutting and cutting allele were measured using Molecular Analyst software (Bio-Rad, Hercules, USA). A ratio of the non-cutting to the cutting allele volumes was calculated as a quantitative measure of LOH in tumour samples.

To verify the sensitivity of the assay for detection of LOH, peripheral blood lymphocytes from a heterozygote and homozygote blood donor were mixed in various proportions to simulate LOH of 0, 10, 25, 50, 75, 90 and 100%. DNA isolated from the cell mixtures was used as a template for the amplification of the *Dra*I polymorphism and LOH in each sample was quantitated by laser densitometry.

### Detection of rearrangements of *Fas*

DNA (10 μg) extracted from 64 tumours was digested with 60 units of the *Taq*I restriction enzyme (New England BioLabs) for 3 h at 65°C. Digested samples were fractionated on 1% agarose gels and blotted onto GeneScreen Plus nylon membranes (DuPont, Boston, USA) under alkaline conditions (Koetsier et al, 1993). The *Fas* gene was detected by hybridization of membranes with a full-length cDNA probe, labelled with [$^{32}$P]dCTP or [$^{32}$P]dATP by random priming (Gigaprime Kit, Bresatec, Adelaide, Australia). The probe was cut out from the pBSAPO-14.2 plasmid (kindly provided by Dr P Krammer) with *Not*I (New England BioLabs). Membranes were hybridized in 50% deionized formamide, 1 M sodium chloride, 10% dextran sulphate, 1% SDS and 500 μg $ml^{-1}$ denatured herring sperm DNA for 16–20 h at 42°C. Membranes were washed to the appropriate stringency and visualized by exposure to Hyperfilm (Amersham, Buckinghamshire, UK) at –80°C.

### Northern blot analysis of Fas mRNA

Frozen colon specimens, from patients who were informative for the LOH assay, were pulverized under liquid nitrogen with a mortar and pestle. The ground tissues were suspended in a lysis buffer containing 4 M guanidine thiocyanate and RNA was isolated using the phenol–chloroform–isopropanol method (Chomczynski and Sacchi, 1987). RNA (10 μg) was electrophoresed through 1% agarose gels containing 17% formaldehyde. RNA was blotted onto GeneScreen Plus nylon membranes

**Table 1** Primers used for the detection of LOH by PCR

| Primer | Sequence | Location |
|---|---|---|
| 1 | 5′-TACAGAAAACATGCAGAAAGC | Exon 7 |
| 2 | 5′-TCAGATAAATTTATTGCCACTG | Exon 8 |
| 3[a] | 5′-CTATTTTTCTTT**T**AAAGGAAAGC | Intron 7 |

[a]Primer 3 contains a mismatch (in bold) to create a control cutting site for *Dra*I.

© Cancer Research Campaign 1998

**Figure 1** Detection of the *Dra*I polymorphism in the *Fas* gene by PCR and restriction enzyme digestion. (**A**) Banding patterns seen in three normal individuals after digestion of a 115-bp PCR product with *Dra*I. M, pUC18 digested with *Hpa*II; 1, undigested PCR product; 2, *Dra*I⁻ homozygote; 3, heterozygote; 4, *Dra*I⁺ homozygote. Unconsumed primer is visible in some samples. (**B**) Loss of heterozygosity in colon tumours of three representative patients (AD, CE, SP). 1, Undigested PCR product; N, normal colonic mucosa; T, colonic tumour. Patients AD and SP show loss of the non-cutting allele and patient CE shows loss of the cutting allele

(DuPont) in 10 × standard saline citrate (SSC) buffer. Northern blots were hybridized with the same Fas cDNA probe used for the Southern analyses, under the same conditions.

### RESULTS

### PCR assay for detection of the *Dra*I polymorphism in *Fas*

A *Dra*I polymorphism, previously reported in the human *Fas* gene (Fiucci and Ruberti, 1994), was used as a restriction fragment-length polymorphism (RFLP) for the detection of allelic losses in colonic tumours. Preliminary attempts to detect the polymorphism, by Southern analysis of genomic DNA digested with *Dra*I, were unsuccessful as no variations in hybridization patterns were observed in a small normal population (data not shown). A PCR-based assay was therefore designed to amplify a smaller region of *Fas* surrounding the polymorphic site. PCR products (115 bp) amplified from normal genomic DNA and digested with *Dra*I revealed either a non-cutting allele of 101 bp or cutting alleles of 61 and 40 bp (Figure 1A).

A *Dra*I site in the *Fas* gene was detected in 64 of 218 chromosomes analysed from a normal population, giving an allele frequency for the polymorphism of 29%. Forty-six per cent of the normal population were heterozygotes and therefore informative for LOH detection. Quantitative ratios of the cutting to non-cutting alleles in normal heterozygotes, calculated densitometrically from the intensity of the DNA bands, varied depending on the gel staining and photography conditions. However, these ratios were consistent within a single gel, so normal and tumour DNA digests were always analysed on the same photograph. The ratio calculated for normal heterozygotes did not vary with the number of PCR amplification cycles or the amount of template DNA used

**Figure 2** Sensitivity of the assay for detection of LOH. PCR products, obtained by mixing PBMNCs of a normal homozygote and heterozygote in the indicated proportions, were digested with *Dra*I to simulate contamination of tumours with normal cells. Densitometric values of the ratios between the uppermost two bands in each track are charted below the figure

**Table 2** Histology of informative colon tumours analysed for LOH

| Tumour differentiation | Informative cases | Number with LOH |
|---|---|---|
| Villous adenoma | 2 | 0 |
| Well | 3 | 0 |
| Moderate | 26 | 3 |
| Moderate/poor | 4 | 2 |
| Unclassified | 3 | 1 |
| Total | 38 | 6 |

**Table 3** Densitometric analysis of samples with LOH

| | Ratio of cutting to alleles | | |
|---|---|---|---|
| **Patient** | **Normal mucosa** | **Tumour** | **LOH (%)** |
| AD | 2.3 | 0.6 | 73 |
| CE | 2.4 | 4.0 | 67 |
| SP | 1.8 | 0.6 | 67 |
| RF | 2.3 | 3.1 | 35 |
| EC | 2.9 | 4.9 | 69 |
| MK | 1.8 | 1.0 | 47 |

© Cancer Research Campaign 1998

**Figure 3** Southern analysis of DNA from normal colonic mucosa (N) and tumours from three individual patients (T), digested with *Taq*I. Filters were hybridized with a 2.5-kb full-length Fas cDNA probe. No alterations in the hybridization pattern are evident in the tumour samples

**Figure 4** Fas mRNA expression in matched normal mucosal (N) and tumour (T) specimens. Northern analysis using a 2.5-kb full-length Fas cDNA probe is shown in the upper panel and ethidium bromide staining of 18S rRNA is shown in the lower panel to indicate RNA loading. Patient TM has similar levels of Fas in both normal and tumour tissue, while the tumours from patients BG and JP show reduction of Fas expression. Patient CE, who had LOH of *Fas*, has no detectable expression in either the normal or the tumour tissue

(data not shown). Lymphocytes isolated from the blood of normal heterozygous and homozygous donors were mixed in various proportions to test the sensitivity of the assay to detect allelic losses in tumours containing normal, uninvolved cells. Analysis of these samples revealed that losses of either the cutting or non-cutting allele of *Fas* could be detected both visually and by densitometry, despite the presence of up to 50% contaminating heterozygous cells (Figure 2).

### Allelic loss in colon tumours

Of the 88 cancer patients involved in the study, 38 (43%) were informative for the *Dra*I polymorphism and exhibited a range of tumour differentiation (Table 2). Six of the 38 informative tumours (16%) exhibited partial allelic loss at the *Fas* locus (Figure 1B), which was confirmed by densitometry (Table 3). Three patients had deletions of the cutting allele; while three lost the non-cutting allele. Because of the small number of patients with allelic loss, no relationship between LOH and tumour stage was apparent. Interestingly, one patient (EC) with LOH in a moderate to poorly differentiated carcinoma had no loss in a benign colonic adenoma removed with the tumour.

### Rearrangements of *Fas*

Normal DNA digested with the *Taq*I restriction enzyme produced three bands when hybridized with a Fas cDNA probe, one of 10 kb, one of 4.2 kb and one of 1.1 kb (Figure 3). No alterations in this banding pattern were observed in 64 tumour DNA samples analysed. There were no apparent allelic amplifications or deletions in any tumour samples, including five of the six patients with LOH analysed by the PCR assay. There was not sufficient DNA available from the remaining patient with LOH for Southern analysis to be performed.

### Expression of Fas mRNA in colon tumours

Fifteen informative colon tumours were analysed for Fas expression, 11 of which had matched normal mucosal RNA samples (Figure 4). All except one sample of normal mucosa examined expressed the 2.7- and 1.9-kb Fas mRNA bands previously reported by Northern analysis (Itoh et al, 1991). Five of 15 tumours analysed expressed Fas at levels similar to that in normal mucosa, however 10 of 15 tumours had reduced or absent Fas expression. There was no evidence of any abnormally sized transcripts in any of the tumour samples analysed.

Four of the tumour samples with LOH had sufficient tissue for RNA extraction and analysis. Of the four patients, only one tumour sample (MK) expressed Fas mRNA. The remaining three tumours did not express any detectable Fas mRNA compared with other samples analysed concurrently. Patient CE had no Fas expression in either the tumour or the normal mucosal specimens analysed. However, a low level of Fas mRNA expression was detected in the tumour RNA after prolonged film exposure.

### DISCUSSION

Apoptosis induced by the Fas antigen is vital for the programmed deletion of immune cell populations and foreign cells. Despite its widespread expression in epithelial tissues, the biological importance of Fas outside the immune system is not fully understood. Reports of abnormalities in Fas protein expression and function in colon tumours and cell lines are now prevalent in the literature and appear to be associated with a failure of apoptotic capacity of the cells. In the present study, we have verified that Fas mRNA is expressed constitutively in normal colonic mucosa, but *Fas* gene transcription was reduced in the majority of tumours analysed. Losses or rearrangements of chromosomes are common in tumour cells (Popescu, 1994) and, when accompanied by an inactivating point mutation in the remaining allele, can abrogate the expression

© Cancer Research Campaign 1998

of tumour-suppressor genes. We found *Fas* gene rearrangements and allelic losses to be rare events in colon cancer, suggesting that other mechanisms are responsible for reduced Fas expression in these tumours.

PCR-based restriction analysis was used to determine the frequency of *Fas* allelic losses in colon cancer. A single base change polymorphism at nucleotide position 836 of the *Fas* cDNA has been reported by Fiucci and Ruberti (1994) with an allele frequency of 33%. The substitution of deoxythymidine for deoxycytidine at this location has no functional significance for the Fas protein, but a recognition site for the *Dra*I enzyme is created. The polymorphism may therefore be used to detect allelic losses of the *Fas* gene in tumours of heterozygotes. Because of its sensitivity, PCR analysis is advantageous for the screening of large numbers of samples, especially for very small pieces of tissue. A common criticism of PCR-based assays for detection of LOH is that formation of heteroduplexes can cause false LOH results, because heteroduplexes are often cut by restriction enzymes less efficiently than are homoduplexes. In the present study, no changes in enzyme cutting were observed, even under amplification conditions that favour heteroduplex formation, including high numbers of PCR cycles and excess template DNA. The sensitivity of the assay was sufficient to detect LOH with up to 75% contaminating normal cells by densitometry, however microdissection of malignant cells from tumours may allow easier visual detection of LOH. This assay may be useful for the detection of LOH at the *Fas* locus in other tumours with abnormal levels of Fas expression.

The allele frequency of the *Dra*I polymorphism determined in this study was similar to that previously determined by denaturing gradient gel electrophoresis of Fas cDNA transcripts (Fiucci and Ruberti, 1994). Loss of heterozygosity was detected at this polymorphic locus in approximately 16% of colonic tumours. This frequency of deletion is similar to that reported by Vogelstein and co-workers (1989) for loss of chromosome 10q in colon tumours, suggesting that the LOH of *Fas* detected in the present study reflects the loss of the entire chromosomal arm. Despite this low frequency of allelic loss, *Fas* gene transcription was reduced in the majority of the informative colon tumours and could not be detected in three tumours analysed with LOH. This suggests that *Fas* deletion is not the only mechanism for loss of *Fas* gene expression. In the tumours of patients with LOH, disruption of the remaining wild-type *Fas* allele by a point mutation or epigenetic mechanisms may explain the complete loss of *Fas* expression.

Analysis of deletion of a gene using a single locus may underestimate the true rate of gene disruption, which can involve discrete segments of the gene. Small deletions in the *Fas* gene have been characterized in autoimmune disorders and lymphomas (Rieux-Laucat et al, 1995; Cascino et al, 1996; Drappa et al, 1996). These deletions remove the signal transduction or 'death' domain of the protein, which is responsible for its cytolytic activity. To determine whether these deletions also occur in colon cancer, we examined Southern blots of tumour DNA samples for altered hybridization patterns. Gross rearrangement of the *Fas* gene was not detected in any of the colonic tumours analysed in this study, suggesting that mechanical disruption or deletion of *Fas* is not responsible for the reduced *Fas* expression in colon tumours. Another study, which examined *Fas* rearrangements by Southern analysis in non-Hodgkin's lymphomas, also reported a low frequency of less than 1%, despite reports of abnormal levels of Fas protein expression in these lymphomas (Xerri et al, 1995). Surprisingly, no *Fas* gene defects or deletions were observed in the four tumours that had exhibited LOH in the PCR analysis. This may be explained by the possibility that the *Dra*I site was lost by a small deletion or a point mutation in the enzyme's recognition sequence. Alternatively, the presence of contaminating normal cells in the tumour samples may prevent the detection of changes in gene dosage by Southern analysis.

The importance of Fas signalling in the development of tumours is not yet known. There have been no reports of spontaneous tumour development in mice with germline mutation or disruption of the *Fas* gene. However, benign expansion of lymphocyte populations and splenomegaly occur in both mice and humans with defective *Fas* genes (Wu et al, 1994; Rieux-Laucat et al, 1995), suggesting that Fas signalling can control the growth of cell populations. When *lpr* mice, which express little Fas protein as a result of a retroviral insertion in the *fas* gene, are crossed with mice that overexpress the oncogene *c-myc*, tumour formation occurs at a greater rate than in the *c-myc*-expressing mice alone (Zornig et al, 1995). Fas deficiency also increases the incidence of B-cell lymphoma in T-cell-deficient mice to 70%, compared with 10% in T-cell-deficient mice with intact Fas protein (Peng et al, 1996). These findings indicate that Fas deficiency has a permissive effect on existing promalignant defects.

During the progression of colon cancer, the ability of colonic epithelial cells to undergo apoptosis in response to mutagenic challenge is compromised. The p53 protein is generally believed to be responsible for induction of apoptosis in response to DNA damage, however loss of p53 is a comparatively late event in colorectal tumorigenesis (Fearon and Vogelstein, 1990). The molecular targets of p53 that carry out the apoptotic signal are unknown, however p53 can bind to the promoter of the *Fas* gene and induce its expression (Owen-Schaub et al, 1995). If Fas is a downstream effector of p53, its loss could directly influence cell death in colonic tumours. However, the role of Fas signalling in the colon and the importance of its loss in colon cancer are still largely unknown.

Fas protein is expressed in variable levels on the surface of several colon cancer cell lines. In some cell lines, low expression of Fas may be up-regulated by the cytokines TNF-α or interferon gamma (IFN-γ) to levels similar to that of activated T cells (Yonehara et al, 1989; Itoh et al, 1991; Moller et al, 1994). Cell lines expressing Fas protein show considerable disparity in sensitivity to agonistic antibodies against Fas. HT29 cells are relatively insensitive, unless preincubated with interferon gamma or protein synthesis inhibitors (Yonehara et al, 1989; Abreu-Martin et al, 1995), while growth of KM12C cells is inhibited by the anti-Fas antibody (Owen-Schaub et al, 1993, 1994). Metastatic variants of KM12C cells are more resistant to the effects of the antibody, despite a similar level of Fas expression (Owen-Schaub et al, 1994). SW620 cells are resistant to Fas ligation, but also express Fas ligand at levels capable of killing T cells, thus avoiding immune deletion (O'Connell et al, 1996). Taken together, these findings suggest that colon tumours may actively avoid immune surveillance and increase their own survival by down-regulating Fas expression or function.

The lack of detectable deletions or other rearrangements in the *Fas* gene in colonic tumours suggests that the gene may be transcriptionally silenced during colon cancer progression. There are recognition sequences in the *Fas* promoter for a number of transcriptional proteins including p53, *c-myb* and NF-KB (Behrmann

© Cancer Research Campaign 1998

et al, 1994; Cheng et al, 1995); the loss of any of which may prevent Fas expression. The promoter region of *Fas* is GC rich and contains a number of CpG dinucleotides. De novo methylation of CpG islands often occurs in tumours and has been associated with epigenetic silencing of tumour-suppressor genes. Future studies in this laboratory will examine the patterns of CpG methylation in the *Fas* gene promoter in colonic carcinomas, as a possible mechanism by which Fas gene transcription is reduced.

### ACKNOWLEDGEMENTS

The authors wish to thank Drs Alex Dobrovic and Andreas Evdokiou for helpful advice during the course of this study. LB is supported by an Australian Postgraduate Award and the Research Foundation of the Queen Elizabeth Hospital.

### REFERENCES

Abreu-Martin MT, Vidrich A, Lynch DH and Targan SR (1995) Divergent induction of apoptosis and IL-8 secretion in HT-29 cells in response to TNFα and ligation of Fas antigen. *J Immunol* **155**: 4147–4154

Adachi M, Suematsu S, Kondo T, Ogasawara J, Tanaka T, Yoshida N and Nagata S (1995) Targeted mutation in the *Fas* gene causes hyperplasia in peripheral lymphoid organs and liver. *Nature Genet* **11**: 294–300

Behrmann I, Walczak H and Krammer PH (1994) Structure of the human APO-1 gene. *Eur J Immunol* **24**: 3057–3062

Cascino I, Papoff G, De Maria R, Testi R and Ruberti G (1996) Fas/APO-1 (CD95) receptor lacking the intracytoplasmic signaling domain protects tumor cells from Fas-mediated apoptosis. *J Immunol* **156**: 13–17

Cheng J, Liu C, Koopman WJ and Mountz JD (1995) Characterization of human *Fas* gene. *J Immunol* **154**: 1239–1245

Chomczynski P and Sacchi N (1987) Single-step method of RNA isolation by acid guanidinium thiocyanate–phenol–chloroform extraction. *Anal Biochem* **162**: 156–159

De Maria R, Boirivant M, Cifone MG, Roncaioli P, Hahne M, Tschopp J, Pallone F, Santoni A and Testi R (1996) Functional expression of Fas and Fas ligand on human gut lamina propria T lymphocytes. *J Clin Invest* **97**: 316–322

Dhein J, Walczak H, Baumler C, Debatin K-M and Krammer PH (1995) Autocrine T-cell suicide mediated by APO-1/(Fas/CD95). *Nature* **373**: 438–440

Drappa J, Vaishnaw AK, Sullivan KE, Chu J-L and Elkon KB (1996) *Fas* gene mutations in the Canale-Smith syndrome, an inherited lymphoproliferative disorder associated with autoimmunity. *N Engl J Med* **335**: 1643–1649

Fearon ER and Vogelstein B (1990) A genetic model for colorectal tumorigenesis. *Cell* **61**: 759–767

Fiucci G and Ruberti G (1994) Detection of polymorphisms within the *FAS* cDNA gene sequence by GC-clamp denaturing gradient gel electrophoresis. *Immunogenetics* **39**: 437–439

Garewal H, Bernstein H, Bernstein C, Sampliner R and Payne C (1996) Reduced bile acid-induced apoptosis in "normal" colorectal mucosa: a potential biological marker for cancer risk. *Cancer Res* **56**: 1480–1483

Hakuno N, Koji T, Yano T, Kobayashi N, Tsutsumi O, Taketani Y and Nakane PK (1996) Fas/APO-1/CD95 system as a mediator of granulosa cell apoptosis in ovarian follicle atresia. *Endocrinology* **137**: 1938–1948

Hall PA, Coates PJ, Ansari B and Hopwood D (1994) Regulation of cell number in the mammalian gastrointestinal tract: the importance of apoptosis. *J Cell Sci* **107**: 3569–3577

Inazawa J, Itoh N, Abe T and Nagata S (1992) Assignment of the human Fas antigen gene (Fas) to 10q24.1. *Genomics* **14**: 821–822

Itoh N, Yonehara S, Ishii A, Yonehara M, Mizushima S-I, Sameshima M, Hase A, Seto Y and Nagata S (1991) The polypeptide encoded by the cDNA for human cell surface antigen Fas can mediate apoptosis. *Cell* **66**: 233–243

Kerr JFR, Winterford CM and Harmon BV (1994) Apoptosis. Its significance in cancer and cancer therapy. *Cancer* **73**: 2013–2026

Koetsier PA, Schorr J and Doerfler W (1993) A rapid optimized protocol for downward alkaline Southern blotting of DNA. *BioTechniques* **15**: 260–261

Leithauser F, Dhein J, Mechtersheimer G, Koeretz K, Bruderlein S, Henne C, Schmidt A, Debatin K-M, Krammer PH and Moller P (1993) Constitutive and induced expression of APO-1, a new member of the nerve growth factor/tumor necrosis factor receptor superfamily, in normal and neoplastic cells. *Lab Invest* **60**: 415–429

Lichter P, Walczak H, Weitz S, Behrmann I and Krammer PH (1992) The human APO-1 (APT) antigen maps to 10q23, a region that is syntenic with mouse chromosome 19. *Genomics* **14**: 179–180 .

Möller P, Koretz K, Leithauser F, Bruderlein S, Henne C, Quentmeier A and Krammer PH (1994) Expression of APO-1 (CD95), a member of the NGF/TNF receptor superfamily, in normal and neoplastic colon epithelium. *Int J Cancer* **57**: 371–377

Muleris M, Salmon R-J and Dutrillaux B (1990) Cytogenetics of colorectal adenocarcinomas. *Cancer Genet Cytogenet* **46**: 143–156

O'Connell J, O'Sullivan GC, Collins JK and Shanahan F (1996) The Fas counterattack: Fas-mediated T cell killing by colon cancer cells expressing Fas ligand. *J Exp Med* **184**: 1075–1082

Owen-Schaub LB, Meterissian S and Ford RJ (1993) Fas/APO-1 expression and function on malignant cells of hematologic and nonhematologic origin. *J Immunother* **14**: 234–241

Owen-Schaub LB, Radinsky R, Kruzel E, Berry K and Yonehara S (1994) Anti-Fas on nonhematopoietic tumors: levels of Fas/APO-1 and bcl-2 are not predictive of biological responsiveness. *Cancer Res* **54**: 1580–1586

Owen-Schaub LB, Zhang W, Cusack JC, Angelo LS, Santee SM, Fujiwara T, Roth JA, Deisseroth AB, Zhang W-W, Kruzel E and Radinsky R (1995) Wild-type human p53 and a temperature-sensitive mutant induce Fas/APO-1 expression. *Mol Cell Biol* **15**: 3032–3040

Payne CM, Bernstein H and Garewal H (1995) Role of apoptosis in biology and pathology: resistance to apoptosis in colon carcinogenesis. *Ultrastruct Pathol* **19**: 221–248

Peng SL, Robert ME, Hayday AC and Craft J (1996) A tumor-suppressor function for Fas (CD95) revealed in T cell-deficient mice. *J Exp Med* **184**: 1149–1154

Popescu NC (1994) Chromosome fragility and instability in human cancer. *Crit Rev Oncogen* **5**: 121–140

Rieux-Laucat F, Le Deist F, Hivroz C, Roberts IAG, Debatin KM, Fischer A and de Villartay JP (1995) Mutations in Fas associated with human lymphoproliferative syndrome and autoimmunity. *Science* **268**: 1347–1349

Rouvier E, Luciani M-F and Golstein P (1993) Fas involvement in $Ca^{2+}$-independent T cell-mediated cytotoxicity. *J Exp Med* **177**: 195–200

Sambrook J, Fritsch EF and Maniatis T (1989) *Molecular Cloning: A Laboratory Manual*. 2nd edn. Cold Spring Harbor Laboratory Press: Cold Spring Harbor, NY

Scott N, Martin I, Jack AS, Dixon MF and Quirke P (1996) Genes mediating programmed cell death: an immunohistochemical study of bcl-2, c-myc and p53 expression in colorectal neoplasia. *J Clin Pathol Mol Pathol* **49**: M151–M158

Sinicrope FA, Ruan SB, Cleary KR, Stephens LC, Lee JJ and Levin B (1995) *bcl*-2 and p53 oncoprotein expression during colorectal tumorigenesis. *Cancer Res* **55**: 237–241

Trauth BC, Klas C, Peters AMJ, Matzku S, Moller P, Falk W, Debatin K-M and Krammer PH (1989) Monoclonal antibody-mediated tumor regression by induction of apoptosis. *Science* **245**: 301–304

Vogelstein B, Fearon ER, Kern SE, Hamilton SR, Preisinger AC, Nakamura Y and White R (1989) Allelotype of colorectal carcinomas. *Science* **244**: 207–211

Wu J, Zhou T, Zhang J, He J, Gause WC and Mountz JD (1994) Correction of accelerated autoimmune disease by early replacement of the mutated *lpr* gene with the normal *Fas* apoptosis gene in the T cells of transgenic MRL-*lpr/lpr* mice. *Proc Natl Acad Sci USA* **91**: 2344–2348

Xerri L, Carbuccia N, Parc P and Birg F (1995) Search for rearrangements and/or allelic loss of the fas/*APO*-1 gene in 101 human lymphomas. *Am J Clin Pathol* **104**: 424–430

Yonehara S, Ishii A and Yonehara M (1989) A cell-killing monoclonal antibody (anti-Fas) to a cell surface antigen co-downregulated with the receptor of tumor necrosis factor. *J Exp Med* **169**: 1747–1756

Zornig M, Grzeschiczek A, Kowalski M-B, Hartmann K-U and Moroy T (1995) Loss of Fas/Apo-1 receptor accelerates lymphomagenesis in EμL-*MYC* transgenic mice but not in animals infected with MoMuLV. *Oncogene* **10**: 2397–2401

© Cancer Research Campaign 1998